# Tailoring Consent to Context: Designing an Appropriate Consent Process for a Biomedical Study in a Low Income Setting

**DOI:** 10.1371/journal.pntd.0000482

**Published:** 2009-07-21

**Authors:** Fasil Tekola, Susan J. Bull, Bobbie Farsides, Melanie J. Newport, Adebowale Adeyemo, Charles N. Rotimi, Gail Davey

**Affiliations:** 1 School of Public Health, Addis Ababa University, Addis Ababa, Ethiopia; 2 Brighton and Sussex Medical School, Falmer, Sussex, United Kingdom; 3 Centre for Research on Genomics and Global Health,National Human Genome Research Institute, National Institutes of Health, United States of America; 4 The Ethox Centre, Division of Public Health & Primary Care, University of Oxford, United Kingdom; Case Western Reserve University School of Medicine, United States of America

## Abstract

**Background:**

Currently there is increasing recognition of the need for research in developing countries where disease burden is high. Understanding the role of local factors is important for undertaking ethical research in developing countries. We explored factors relating to information and communication during the process of informed consent, and the approach that should be followed for gaining consent. The study was conducted prior to a family-based genetic study among people with podoconiosis (non-filarial elephantiasis) in southern Ethiopia.

**Methodology/Principal Findings:**

We adapted a method of rapid assessment validated in The Gambia. The methodology was entirely qualitative, involving focus-group discussions and in-depth interviews. Discussions were conducted with podoconiosis patients and non-patients in the community, fieldworkers, researchers, staff of the local non-governmental organisation (NGO) working on prevention and treatment of podoconiosis, and community leaders. We found that the extent of use of everyday language, the degree to which expectations of potential participants were addressed, and the techniques of presentation of information had considerable impact on comprehension of information provided about research. Approaching podoconiosis patients via locally trusted individuals and preceding individual consent with community sensitization were considered the optimal means of communication. Prevailing poverty among podoconiosis patients, the absence of alternative treatment facilities, and participants' trust in the local NGO were identified as potential barriers for obtaining genuine informed consent.

**Conclusions:**

Researchers should evaluate the effectiveness of consent processes in providing appropriate information in a comprehensible manner and in supporting voluntary decision-making on a study-by-study basis.

## Author Summary

Informed consent to biomedical research in developing countries is a highly topical issue. When consent forms and processes are simply borrowed from developed countries, obtaining genuine informed consent becomes extremely challenging. This paper examines how a quick and relatively simple intervention (Rapid Assessment) can influence the design and implementation of informed consent processes in the context of biomedical research involving poor, socially stigmatized and illiterate communities in a developing country. The paper goes on to discuss the effect of social, cultural, and economic factors identified by the intervention in a particular context and demonstrates how knowledge of these influences helped to develop a socially relevant and practical consent process prior to conducting a programme of community-based genetic research. The paper concludes that this intervention is an effective and economical means by which to ensure the efficacy and ethical integrity of consent processes when recruiting participants to new research sites, even within countries with which researchers are already acquainted.

## Introduction

Informed consent is a fundamental prerequisite for undertaking ethical research. Marshall states that informed consent is influenced by a number of factors including ‘the cultural setting of the research project and local beliefs and customs, the nature and goals of the study, communication issues that affect comprehension of information, and discrepancies in social and economic power between researchers, sponsors, and individuals and communities’ [1].

These issues intensify when externally sponsored research is conducted in developing countries. Firstly, the information sheets and consent forms borrowed from developed countries may contain technical concepts that are not familiar to research participants in developing countries [2],[3]. Secondly, the use of written information sheets and consent forms may be inappropriate in places where research participants are not literate. Thirdly, in some communities the norms of decision-making do not emphasize autonomy at the individual level. It may be culturally inappropriate to approach individuals to participate in research before obtaining permission from community leaders, elders or tribal chiefs. In other cases a male head of a family is expected to consent to research on behalf of his wife and adult children [4],[5]. Fourthly, it may be difficult to judge whether provision of health care constitutes an undue inducement for participating in research. Finally, participants' trust in a research or other institution may override their ability to make genuinely autonomous decisions [4].

Several sets of guidelines have been developed to deal with these difficulties. These recommend preceding individual consent with community level consultation and approval [2],[3],[6]; using verbal consent instead of written consent in countries where the majority of research participants are not literate [2],[7],[8]; applying other innovative forms of documenting the individual informed consent process such as audio or video tape instead of signatures [4], [9]–[11]; and providing information about the study at the correct level of comprehension [10],[11].

The issues and concerns discussed above point to the need for detailed understanding by researchers of the social and cultural context of the community and potential research participants before informed consent is sought. We assessed determinants of and approaches to gaining informed consent for biomedical research in a predominantly rural Ethiopian population, and discuss the practical ways in which we used this information in a subsequent genetic study. This rapid assessment was undertaken prior to a study (referred to later in this manuscript as ‘the genetic study’) investigating the genetic basis of susceptibility [12] to podoconiosis (non-filarial elephantiasis) [13] using data from affected sibling pairs, their biological parents and unaffected controls in southern Ethiopia.

## Methods

### Ethics statement

Ethical approval for conducting the rapid ethical appraisal was obtained from the ethical review board of the Faculty of Medicine, Addis Ababa University and the Ethiopian Science and Technology Agency. Informed oral consent was obtained from the study participants before conducting the interviews and discussions. The use of oral consent was approved by the ethical review boards because the majority of the study participants cannot read and write. All consent process was documented in a tape record.

### Study setting

The study was conducted in Wolaita Zone, 390 kms southwest of Addis Ababa, the capital city of Ethiopia. It covers a total area of 4541 sq. kms and has 1.6 million inhabitants. The majority of the population earn their livelihood from crop production (61.3%) followed by livestock rearing (22.3%). Prolonged contact with irritant volcanic soils is common, and underlies the high (5%) prevalence of podoconiosis in the Zone [14]. Local vernacular terms for podoconiosis include *gediya kita* in Wolaitigna and *ye'zihone beshita* in Amharic.

The Mossy Foot Treatment and Prevention Association (MFTPA) is a local non-governmental organization (NGO) in Wolaita Zone which has worked on prevention and treatment of podoconiosis since 1998. Currently, it has 15 outreach sites and treats more than 30,000 patients per year. Each outreach site is staffed by two fieldworkers: one health service provider and one social counsellor. The MFTPA is well known through Wolaita Zone as it is the only organization providing treatment and care of people with podoconiosis. It has built up an enduring relationship with the community through prevention activities in schools, churches and mosques, and through provision of social rehabilitation to patients through vocational skills training and microcredit schemes.

### Study design

The study employed qualitative methods - focus-group discussions (FGDs) and in- depth interviews (IDIs). The study was conducted using semi-structured interview guides for IDI and FGD adapted from a rapid assessment method validated in The Gambia [15]. Five types of interview guides were prepared: four IDI guides each for researchers, fieldworkers, community members and *kebele* (the smallest administrative unit/village in Ethiopia) heads; and one FGD guide for discussion with community members. The interview instruments were prepared in English, translated into Amharic or Wolaitigna as appropriate and back-translated into English to check for consistency. We found high consistency in the majority of the translations. A major inconsistency was while different Amharic terms were used for podoconiosis, the Wolaitigna translation resulted in only one term, *gediya kita*. Through discussion with local people, we realized that *gediya kita* is derogatory to patients, and some of the respondents preferred to use different terms that mean leg swelling more generally. Pilot testing of the instruments was done with one researcher in the United Kingdom and a fieldworker and community member in Wolaita.

### Sampling and study subjects

Generally, sampling was purposive based on pre-defined inclusion criteria for enrolling participants. IDIs and FGDs were conducted until no new relevant ideas emerged from further interviews or discussions. The study targeted four groups of participants. The first group incorporated scientists and researchers that had experience working in Wolaita Zone on genetics or other biomedical studies. IDIs were conducted with four scientists and researchers in this phase of the study. The second group included trained MFTPA fieldworkers: three social and counselling workers and four health workers. The third group involved IDIs with (i) two individuals involved in the administration and coordination of the activities of the MFTPA, (ii) two heads of *kebele* and (iii) two community leaders. The fourth group comprised community residents of both sexes and included patients and healthy subjects. In total 32 community members participated, 8 in IDIs and 24 in FGDs.

Overall, 19 females and 27 males participated in this study. Half of the community FGD participants, half of the researchers and one of the fieldworkers interviewed were females. All interviewees from MFTPA management and *kebele* offices were males. The age of the respondents ranged between 23 and 70 years. The educational status of the fieldworkers ranged between early secondary level and college level education. Most of the community interviewees had had no formal education.

### Data collection

With the exception of the community interviews, data were collected by one of the principal investigators. An experienced Masters in Public Health graduate who speaks Wolaitigna (the local language) did the IDIs and moderated the FGDs with community members. Before conducting the IDIs and FGDs, he was trained about the purpose of the study, the data collection instruments and interviewing techniques.

### Data analysis

Audiotapes were transcribed anonymously, and interviews conducted in Amharic and Wolaitigna were translated into English and imported into OpenCode software v.2.1 (a freely available computer program for managing and analyzing text data) [16]. Open coding was used to identify themes that were developed into conceptual categories. Data were iteratively examined to identify additional themes. The issues that arose fell into the following primary thematic domains: basic knowledge of the community about the concept of research; language and content of information provided; comprehension of information; opinions of the study participants about the notion of informed consent; motivations for consenting to participate in research; decision-making processes; and preferred approaches and communication styles with the community.

## Results

### Language levels in information sheets and consent forms

The interviewed researchers stressed that consent forms and information sheets that are requested by institutional and national ethical review committees in Ethiopia and in funding countries are tailored to Western populations. They stressed that the language used in such forms often requires a high level of technical understanding and is based on models emphasizing individual autonomy.

Most community participants confirmed that they could not read information sheets and consent forms. They also stated that they found it difficult to understand even when researchers read written consent forms line by line without further explanation.

Even if I am a grade four student, I still can't read. So I prefer verbal information to written one. [Female community participant]

They read out [the information sheet] line by line, we know they do that for us… hence we pay less attention towards it…. whatever they needed we say okay… and it is difficult for us to understand a formally written material being read out. [Female community participant]

### The role of information in the consent process

People in rural Wolaita are not used to receiving information for individual decision-making. Most participants did not understand that information provided prior to consenting was offered as a guide for them to decide whether to participate, but thought it was provided as a form of health education. This was demonstrated when they described the information they had received during their participation in previous research. They expressed the information process in the following ways: ‘*they taught me*’, ‘*they educated me*’, ‘*they advised me*’.

As a result, researchers and fieldworkers suggested identifying and building on words, narratives and metaphors used in the community to provide information to assist understanding of the proposed research.

You have to work out how to get across some of the concepts like ideas about gene, heritability and things like that…. I think we have to discuss the concepts without using the technical terms. [Researcher]

You should talk to data collectors in detail. There is no need to use medical/biological jargon. You should use simple terms, and also important to clarify every important aspect of the purpose of the study. [Researcher]

The solution for this is to use the language of the individual. The second thing is to correlate the information provided with some examples. This helps to increase understanding, as the community is not very literate. Then, one should conclude his information provision by revising the most important points and by asking questions. [Fieldworker]

Besides oral communication, one researcher suggested that the use of pictures, videos, diagrams and other descriptions could help to minimize loss of information, as most people are not literate.

### Content of information

Participants said that information provided to them before enrolling into research was valuable, and saw the provision of information as a form of respect for prospective research participants. During consent processes, information should be provided on topics that are of most interest to participants. In this study prospective participants considered the most important information to know was the expected benefit of the research. Fieldworkers consequently stressed that the consent process should focus on the potential benefit of the proposed research to the participant and/or the community.

The most important thing is to explain that the purpose of the study is to benefit the community. In Wolaita there is a proverb, ‘For its own benefit, a shovel cuts *kocho*’*. [Fieldworker] *Part of staple diet prepared from roots of false banana tree

In addition, the fieldworkers and patients indicated that people want to know who the researchers are; why the study is proposed; how confidentiality will be preserved; that findings will not enable identification of either family or individual; and the arrangements for communicating findings of the study with the community. A number of former research participants complained that they have not been given feedback from previous studies.

Researchers and fieldworkers suggested that information provided during the consent process should incorporate issues about the general purpose of research, its aim to improve understanding of a given condition, and its role in the discovery of new technologies. In addition, they suggested discussing the difference between research and medical care in simple and clear terms.

[Explain that] you are here to conduct a study, and not to provide clinical care or treatment. You should also explain that there is aggregation of the disease in some families and that you intend to know whether there are familial factors. [Fieldworker]

[You should tell them by saying] We are here to conduct a study that investigates a factor in the ‘blood’ that risks people for podoconiosis, and the results could potentially help to advise susceptible people to take precaution. [Researcher]

### Understanding of the community about research

The role of research in increasing knowledge about a given condition was not understood by most participants from the community. Most patients and some fieldworkers used the words *xinatia* (research) and *mermerechiyowga/taletiyowga* (clinical diagnosis) interchangeably. Community members assume that collection of samples is intended for clinical diagnosis, particularly when they participate in biomedical studies within a health care unit. As a result, some participants complained that they have not yet received previous ‘results’ on an individual basis from research studies. The high level of illiteracy in the population was unanimously mentioned as one reason for confusion, lack of interest and low level of understanding about research. The fieldworkers and MFTPA staff also noted that patients' knowledge about research depends on the extent of explanation they received during their participation in research.

### Approaching the community

Researchers and fieldworkers suggested that the consent process should start by developing a relationship with the prospective participants. Issues considered important to address during this phase are: understanding the interests of the community; recognizing the major problems (health or non-health) of the community; targeting those problems and showing a desire to help address them; and being polite and showing respect to the culture, religion and livelihood of the community.

We should not make the issues strong, we should politely and with love discuss with them smoothly. As a man of God we have to visit their houses and chat with them about their health problems, life puzzles. Or whatever they might have. [Fieldworker]

All groups of participants advised that guest researchers should approach podoconiosis patients through reputable local intermediaries like the MFTPA fieldworkers because the fieldworkers are well respected.

### The community in the consent process

In Wolaita, it is uncommon for people to receive individual-based information in a one-to-one discussion. Local community gatherings, group discussions and consultations are the usual modes by which people receive information. Moreover, some people wish to consult with their neighbours, colleagues and partners before making a decision, because of their communal living style. Researchers and fieldworkers stressed that community sensitization and group information provision should precede information provision for consent at individual level. They indicated that community sensitisation could be used to educate patients about research, to relay information about the purposes of the proposed research, its difference from clinical care and any potential benefit to the community. Community sensitization is likely to clear doubts, making participants more receptive when data collectors visit their households. It may also act as a forum in which the method of selection may be explained, avoiding later confusion when only some households or individuals are approached during the study.

### Ability to make voluntary decisions

Participants unanimously agreed that patients welcome research conducted in collaboration with MFTPA. However, it was not clear whether patients knew they could refuse to participate in a study without being denied routine services from the MFTPA. Fieldworkers and some researchers indicated that research with monetary compensation might induce podoconiosis patients to participate because they are generally poor. As a result, they stressed that ensuring ‘*kelib yehone simiminet*’ (‘true voluntariness’) should be part and parcel of the consent process because willingness to participate *per se* does not guarantee that genuine consent has been obtained. They also noted that true voluntariness should be seen in terms of offering the participant appropriate and full information, establishing that a participant understands the relevant issues, and confirming that a participant has consented without undue pressure from other people, including the MFTPA and researchers.

One approach is to use carefully formatted questions that test comprehension of voluntariness. Researchers suggested that the following points should be included in such questions: whether the individual has understood the difference between treatment and research; that he/she can decline participation without negative consequences such as being denied healthcare to which they are otherwise entitled at the MFTPA or other government health facilities; that he/she can choose whether or not to participate; and that the benefits he/she gains by agreeing to participate are limited.

## Discussion

This rapid assessment exposed a number of issues surrounding the informed consent process, and helped to address the gap between participants' expectations and issues thought to be essential by researchers and ethical review boards. The findings reflect the need to develop the informed consent process from a cultural perspective and indicate ways of improving comprehension of the process in a low-income setting.

We found that participants supported the concept of informed consent as a requirement for ethically sound research. They valued the information provided and the respect and politeness shown to them by researchers. They also stated that the content of the information must be focused around patients' perspectives and expectations. A central component of information provision during the consent process is the ways in which information is provided. Information provision at individual level is not common in the Wolaita community. Other approaches of tailoring the communication style to the local context are essential [2]. Respondents advised preceding the individual level consent process with group information provision using community gatherings. Other researchers also advocate such pro-active community-based information giving in an African context [3],[6]. It is essential to design institutional and cultural practices to promote comprehension [17].

We subsequently used the findings from the rapid assessment to inform the consent process for the genetic research. We began by preparing sensitization meetings about the genetic study to which fieldworkers who represent their local community were invited. A comprehensive discussion was conducted with the fieldworkers about the purpose of the research and the need to have community discussion and dialogue before processing individual level consent. Administrative staff of the MFTPA conducted reiterative community sensitization discussions and consultations. The main topics discussed included aims of the research, how information was to be kept confidential, and the right of the family to withdraw from the research.

Further discussions were conducted with church leaders, elders and local administrative officials who also participated in the process of recruiting participants. After we ensured that a general consensus had been reached at community level about the acceptability of the research, fieldworkers approached eligible families in their local areas and held family-level discussions in the presence of the head of the family, eligible participants in the family and most other members of the household. Discussions focused on why the family was selected, and whether they were willing to be approached by the research team. Families willing to be approached for further enquiry were given an appointment and were visited by the research team. The research team explained the research in more detail and asked if the family was willing to take part, generally confirmed by the head of the family. Finally, each eligible study participant (affected siblings and their parents) were asked for independent individual-level consent.

Another factor identified in the rapid assessment as important in the consent process was the language used in relaying information. Lack of education and access to scientific concepts in biomedical research by potential participants is well known as a challenge to informed consent process in developing countries [2],[3]. Careful tailoring of words and concepts to the perspective of research participants has been suggested as an alternative to technical terminology [18], while some researchers recommend the use of pre-determined and rehearsed stories [19]. In countries where there is no clear terminology even for the word ‘research’, it may be difficult for participants to relate the information given to them about a study to the concept of research [3].

Participants in this study had difficulty distinguishing between information given to guide their decision about participation and more general health education. To overcome this issue in the genetic study, we utilized the community sensitization sessions to educate the community about research using findings of previous research in which the community participated. Because of the prevailing belief that podoconiosis is a genetic disease in the community, we used locally used terms like ‘passed from parents and grandparents’ and ‘blood’ to express genetic occurrence of disease.

Most participants in this study did not favour the use of written information sheets and consent forms. In contexts like ours where the study subjects are illiterate, our findings corroborate others' favouring oral consent as an appropriate alternative to written consent [2],[7],[8]. The use of written consent forms in less literate populations may unfairly exclude potential participants who cannot read or write, and may create confusion and anxiety particularly for potential participants who are unaccustomed to signing documents [2]. Our study participants indicated they were not comfortable with the approach whereby data collectors read a consent form line by line. The experience of other investigators is that understanding is improved through use of a conversational style instead of reading an information sheet [2].

We found ourselves caught between two sets of expectations - that of the Wolaita community who favoured verbal approach to information delivery and indicating consent, and that of the study funders and Western collaborators, who required detailed written information sheets and written confirmation of consent. We therefore developed detailed information sheets, but also developed standardised methods of verbally explaining in the community sensitization meetings. We obtained verbal consent (approval) from the community and its leaders who expressed their interest in participation after thorough discussions. Family level consent was also verbally indicated when every adult member of the family agreed that the family can be part of the study. Individual-level consent, however, was confirmed by written consent through which every participating individual showed agreement to participate by signing or thumb-printing on the consent form.

Information provided during the consent process should target the expectations of participants and try to solve their misconceptions. Our respondents indicated that the most important information they wished to receive was the expected therapeutic benefit of the study. Generally, most participants did not differentiate between the purposes of biomedical research and those of medical care. Lack of understanding about research was evidenced not only among the research participants but also among the fieldworkers. This may indicate the existence of the ‘therapeutic misconception’ [6], [20]–[23], but may also reflect the desire podoconiosis patients have to find an effective treatment as an outcome of innovative research. This lack of ability to differentiate research from medical care is common and suggests that researchers must go to some lengths to clarify the difference between the two to protect participants and encourage consistent community involvement in future biomedical research.

Following the rapid assessment, we trained fieldworkers of the MFTPA, emphasising the limited contribution the genetic study would have in relation to immediate or future therapeutic benefit by explaining that it was the beginning of a long journey of understanding about the disease. We also conducted community sensitization to improve participants' awareness that no therapeutic benefit was guaranteed by the study. Managers of the MFTPA and trained fieldworkers had frequent discussions with patients about these topics during routine clinic visits.

Patients also expected to receive non-clinical benefit when participating in a study. Discussions conducted with the community, fieldworkers and managers of the MFTPA enabled us to arrive at a package of compensations that addressed participants' expectations without providing an undue inducement to participate. In the genetic study we compensated for the time participants devoted to the study by offering them a package of items (socks, bleach and soap) useful for keeping their feet clean.

Other information deemed important by international ethical guidelines: the purpose of the research; who the researchers are (their institutional affiliations); what will be done with any biological sample; provisions to maintain confidentiality; whether and how participants will be told the findings of the study, were also considered important in the consent process by community respondents, fieldworkers and researchers. We therefore included this information in both the written and the verbal explanations of the genetic study.

Community respondents emphasized the high degree of trust placed in the MFTPA, and the way this might influence a decision to participate in research linked with the MFTPA. Placing trust in an individual or organization does not obviate making an autonomous decision; it may simply represent another factor weighed by a potential participant when making a decision. Trust and feeling of mutual responsibility among research participants and collaborators can establish a fertile ground for a sensible and effective informed consent process [24].

Several factors emerged as possible constraints to making a voluntary decision in this rural Ethiopian setting: low income (and hence low access to even simple materials to assist with treatment of podoconiosis); poor access to health care; and a traditionally hierarchical decision-making structure in which responsibility for decision-making was frequently vested with leaders of communities and families. Conversely, patients associated the MFTPA with provision of materials; provision of health care and empowerment of individual patients. We therefore felt it was important to stress that health care would not be affected by the patient's decision, and in particular that the patient's relationship with the MFTPA would not be altered by a decision not to take part in research.

The genetic research involved children (sibling pairs) and women (mothers of children). It is difficult to prove whether participation of these individuals was really voluntary in a community in which the family structure gives more say to the husband, who is usually the head of the household. In our study, we recruited only those aged at least 18 years (the legal age for giving consent in Ethiopia) to promote adult children's decision-making capacity. However, the tension between respecting the traditional decision-making pattern and promoting individual consent to research is complex in this and similar contexts [2] and would benefit from further research.

This research indicates that language, content and delivery of information, route of approach to the community and preliminary sensitization of the community are important factors to take into account when seeking to design a consent process which supports prospective participants' ability to make an informed decision about participation in research in this setting. Approaching potential participants under the auspices of an organization well known to the community need not compromise free decision making, and may be preferred by participants in some contexts.
